# The British Pharmaceutical Conference

**Published:** 1918-07-27

**Authors:** 


### The British Pharmaceutical Conference.

The annual meetings of the British Pharmaceutical Conference during the years of war have been more or less formal affairs; original papers on pharmaceutical topics which, in ordinary times, are contributed at the annual meetings are hardly expected at the present time, since most of the best brains in pharmacy are engaged in problems of more immediate national importance. Nevertheless it is desirable that a valuable institution like the British Pharmaceutical Conference should be kept together, and this is the purpose of the formal yearly functions. The fifty-fifth annual meeting was held at 17 Bloomsbury Square, London, on July 10, when the President, Mr. C. A. Hill, delivered an interesting address in which he took stock, so to speak, of our drugs—a task which no one is more capable of doing than he. It is satisfactory to know that most of the essential medicinal products have been, up to the present, available in sufficient if not abundant quantities, although there are a few vegetable drugs which either are unobtainable or are in quite small supply. For instance, storax, hellebore, stramonium, and uva ursi, which we used to get from Austria, cannot now be bought, but fortunately there is a limited quantity of English stramonium available; scammony, opium, and tragacanth cannot, of course, be obtained from Turkey, but there are sources of supply. Most of the drugs from the Persian Gulf, like asafetida and galbanum, are difficult to find nowadays, while bulky vegetable drugs (like cascara sagrada and sarsaparilla, for instance) which come from overseas are likely to become scarcer and scarcer. Calumba root, which used to sell in the open market at 17s. per cwt., now fetches more than 200s. when any of it is offered. Gum benzoin fetches extravagant prices, and the freight question largely accounts for the extremely high value of honey. Indeed, freight and insurance are responsible for the costliness of many drugs; to illustrate this, Mr. Hill mentioned that a consignment of Mexican scammony root was landed a few weeks ago the invoice value of which was £1,000 at the port of shipment, while the freight and charges amounted to £1,800. The cultivation of vegetable drugs at home, which The Hospital has endeavoured to encourage, has met with a very considerable measure of success, and the President was able to say that the home production of colchicum, digitalis, poppy-heads, belladonna, and hyoscyamus was practically sufficient to meet the requirements of this country.

